# Exploring the impact of display types of information about autonomous driving in semi-autonomous vehicles on drivers’ situation awareness and take-over performance under different driving scenarios

**DOI:** 10.1371/journal.pone.0329760

**Published:** 2025-08-22

**Authors:** Chengmin Zhou, Yuxuan Luo, Jake Kaner

**Affiliations:** 1 College of Furnishings and Industrial Design, Nanjing Forestry University, Nanjing, Jiangsu, China; 2 Jiangsu Co-innovation Center of Efficient Processing and Utilization of Forest Resources, Nanjing, China; 3 School of Art and Design, Nottingham Trent University, Nottingham, United Kingdom; Southwest Jiaotong University, CHINA

## Abstract

With the advent of the era of autonomous driving, designing an effective and appropriate autonomous driving information display is crucial for ensuring driving safety. Head-up Display (HUD) is regarded as a promising way for presenting in-vehicle information in the future. This study conducted a simulation experiment to explore the impacts of three types of autonomous driving information displays on HUD on Situation Awareness (SA) and take-over performance, while considering the complexity of different driving scenarios. The experiment used in this study adopted a Latin square experimental design and employed an integrated eye-tracking technology with self-reporting and the Situation Awareness Global Assessment Technique (SAGAT). The results show that although young drivers perform better with the Augmented Reality (AR) display in various complex scenarios, particularly in high-complexity scenarios (the fixation duration with AR display was significantly shorter than that with Pseudo-3D (P3D) display; P = 0.012). However, the advantages of the AR display will weaken as the complexity of the scenarios decreases. Additionally, the Surround Recognition (SR) display is more likely to reduce drivers’ SA (the fixation counts on the SR display was significantly higher than that on the P3D and AR displays; P < 0.001) and take-over efficiency (the take-over reaction time for the SR display was significantly longer than that for the AR display; P = 0.09), especially in medium-complexity scenarios. Meanwhile, male participants pay more attention to the autonomous driving information on HUD. Nevertheless, there is no obvious difference between males and females in terms of specific preferences for the types of displays. The results of this study are expected to provide some inspiration for the design of autonomous driving information on HUD.

## Introduction

With the large-scale application of artificial intelligence and 5G networks, product automation is revolutionizing various fields. Human-computer interaction in modern electric intelligent vehicles is primarily challenged by complex driving tasks [1,2]. Research has shown that autonomous driving technologies can effectively reduce accidents caused by driver error [3].

Currently, the autonomous driving technology system can be divided into two major technical paradigms, namely the basic type and the enhanced type, according to the SAE J3016 standard. Basic autonomous driving covers levels from L1 (Driver Assistance) to L2 (Semi-autonomous Driving), and its technical characteristics are manifested as single-dimensional control capabilities. For example, Adaptive Cruise Control (ACC) for longitudinal control, Lane Keeping System (LKS) for lateral control, and Automatic Emergency Braking (AEB) for safety intervention. Through the function combination strategy (e.g., ACC + LKS = Lane Centering Control, LCC), such systems can achieve coordinated lateral and longitudinal control in specific scenarios [4]. Enhanced autonomous driving focuses on the technological evolution at the L2+ level, serving as the core form of the transition from L2 to L3 (Conditional Autonomous Driving), but it is still at the semi-autonomous driving level. In essence, it is a combination of multi-modal environmental perception and pre-set domain control capabilities. Relying on high-precision maps or computer vision algorithms, it can achieve complex functions such as automatic lane changing and autonomous overtaking in limited scenarios within the Operational Design Domain (ODD) like highways [5]. Advanced Driver Assistance Systems (ADAS), as a representative of L2+ autonomous driving, have developed rapidly in the past decade and are becoming increasingly popular worldwide [6,7].

Existing research has generally demonstrated that autonomous driving technologies can acquire information about changing environments more efficiently than humans and demonstrate rational driving behaviors [8,9]. Nonetheless, there is still a lack of consumer trust in autonomous driving technology, as evidenced by the volume of user data and the numerous reports of L2+ assisted driving malfunctions from different automakers [10]. Therefore, manual driving involvement must still be taken into account in contemporary semi-autonomous driving system designs [11,12]. In the design of the Human-Machine Interface (HMI) for L2+ semi-autonomous electric vehicles, it is essential to adopt a multi-modal information presentation strategy to ensure that drivers maintain sufficient Situation Awareness (SA) capabilities during the handover of vehicle control [13]. Research shows that in the lower levels of automated driving, the driver should maintain maximum focus on the road and that Head-up display (HUD) is the most effective means of providing information [14]. With the advancement of technology, electric vehicles are increasingly utilizing various in-vehicle HUDs, including the Widescreen Head-Up Display (W-HUD) and the Augmented Reality Head-Up Display (AR-HUD). It’s becoming common practice in new-generation electric cars in the Chinese market to show various autonomous driving-related data through the HUD.

The core differences between the W-HUD and theAR-HUD lie in information presentation and spatial depth. The W-HUD projects static driving information (eg., vehicle speed, navigation prompts) to a virtual image distance (VID) of 2.5 meters via optical reflection. Its technical characteristics are limited by the field of view (FOV) and the static nature of the displayed content [15]. In contrast, AR-HUD leverages waveguide technology and AR algorithms to achieve dynamic 3D projection at a viewing distance exceeding 10 meters, directly fusing navigation, warnings, and other information with the real driving environment.

SA was first employed to evaluate the proficiency of machine operators, but it is now acknowledged as a crucial factor in the automobile industry, specifically in autonomous driving technologies. It offers valuable insights into the driver’s condition and driving proficiency [16]. In dynamic driving operations, SA has a significant impact on human decision-making processes. It requires comprehensive, accurate, and real-time information acquisition by the driver about the surrounding driving environment and situation, including the process of acquiring the elements of the environment (SA level 1-SAL1), understanding them (SA level 2-SAL2), and making predictions about their future state (SA level 3-SAL3) [17,18]. SAL3 is the highest level in a hierarchical arrangement of these three levels. The SA system has the characteristic of dual subjects, encompassing both the human perception dimension of the driver and the machine perception dimension of the intelligent system [19]. The system’s and the driver’s respective SA skills will trade off as automated driving technology advances. Most electric intelligent vehicles include L2+ level automated driving technology, which requires constant SA exchanges between the driver and the system in order to achieve secure and productive cooperative driving. Designing visual signals is a frequent strategy to assist drivers in regaining and maintaining SA [20]. For instance, the layout of the display system, which provides spatial information about the car and its surroundings, improves the driver’s sense of awareness [21]. Almost all electric intelligent vehicles equipped with autonomous driving functions have visual displays of autonomous driving information. However, if the provision of additional information leads to a decrease in the driver’s SA capabilities, it may be detrimental [22]. Thus, more investigation is required to determine how autonomous driving information display types affect driver safety.

The main function of the autopilot take-over task is to initiate a Take-over Request (TOR) from the system, asking that the driver resume control of the vehicle in the event of a real-time driving situation outside the ODD or for reasons determined by the autopilot system itself. The driver’s SA capacity to determine the next course of action is also greatly influenced by the objective road circumstances. Traffic density, weather, and road conditions are objective driving environment characteristics that might affect a driver’s driving take-over performance [23]. While driving in high-density environments and inclement weather can make it more difficult for drivers to react and maneuver, they can also make it more common for them to perform braking, deceleration, and lane changes. The influence of traffic environment characteristics on the efficiency of driving take-overs presents a non-linear two-way regulatory effect. This shortens the time between an object’s proximity to another vehicle and the driver’s reaction to the operation [24]. Furthermore, driving environments that are excessively basic and monotonous might also impair drivers’ ability to drive safely [25]. This is why it’s critical to consider the possibility of reduced cognitive function associated with repetitive driving tasks. Studying how exterior factors and the autonomous driving information display on the HUD interact to influence driver take-over responses in a brief amount of time is critical.

This study integrates existing research paradigms to establish an evaluation system for autonomous driving information display types on HUDs, incorporating scenario complexity. It focuses on determining the effects of different existing HUD autonomous driving information display types on young drivers’ SA ability under multilevel complexity driving scenarios and their effects on their take-over performance under multilevel complexity take-over scenarios. The study addresses two scientific questions: 1. What type of autonomous driving information display is helpful for enhancing the driver’s SA ability and take-over ability? 2. Will the advantages and disadvantages of different autonomous driving information display types change according to the variation in the complexity of the driving scenarios?

Based on an eye-tracking technique and SAGAT questionnaire reports, respectively, an experiment was carried out to examine the roles of different display styles in situations of various complexity. The objective of this approach is to provide empirical evidence for optimizing existing autonomous driving information displays on HUDs and offer recommendations for the development of such displays.

## Methods and process

### Display the type of automatic driving information

**Fig 1:**
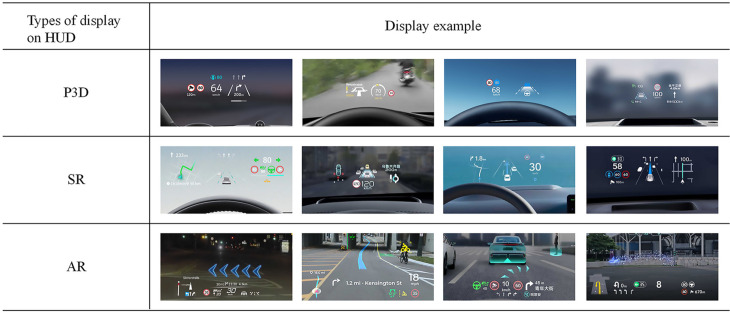
Three display types of autonomous driving information on the HUD in the Chinese vehicle market.

This study concentrates on determining the significant impact of the design of autonomous driving information on the HUD on drivers, rather than evaluating the specific design of the display of autonomous driving information. The three categories of information displayed on the HUD are selected from popular automobile brands in the Chinese market according to different display types of autonomous driving information, which mainly include environmental information and vehicle information display. The first type of display scheme is the Pseudo-Three-Dimensional (P3D) spatial perception based on the W-HUD. With the application of high-definition sensor fusion technology, the automatic driving information presented on the W-HUD has realized the intuitive mapping of key road conditions, from a P3D display without a car (e.g., NIO ES6) to a P3D display with a car (e.g., AITO M5). The second type is to project Situational Recognition (SR) autonomous driving information onto the HUD. With the development of real-time rendering technology, SR interface displays are widely adopted in the current Chinese automotive market. Compared with the traditional P3D information presentation, details such as the body outline and turn signal flashing on the SR interface are clearly visible (e.g., Li L9). Even road directions, traffic signs, and even potential traffic conflict points are visualized to the driver (e.g., ONVO L60). The third type is autonomous driving information designed on AR-HUD. AR technology can seamlessly integrate virtual navigation guidance, vehicle status, and surrounding environment perception information into real road scenes (e.g., Panasonic CES 2021). Meanwhile, more and more manufacturers are leveraging AR technology to make navigation and other information more entertaining (e.g., AITO M9). Fig 1 lists the three display types of autonomous driving information on the HUD in the Chinese vehicle market.

**Fig 2:**
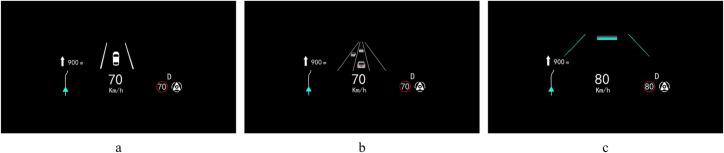
Displays types of autonomous driving information: a) P3D display on the W-HUD; b) SR display on the W-HUD; c) display on the AR-HUD.

To minimize the impact of the differences in autonomous driving functions on the driver’s SA and take-over performance, the variables of autonomous driving functions are fixed according to the existing display types of autonomous driving information on the HUD. This study only takes into account vehicle interfaces with AEB, ACC and LKA functions. We have summarized and designed three different HUD interfaces as shown in Fig 2.

Type A (P3D) is the automatic driving information presented on the W-HUD in P3D graphics, mainly static display of lane lines and self-driving vehicles.

Type B (SR) is the SR interface projected onto the W-HUD to dynamically display multiple lane lines, autonomous vehicles, and other traffic participants.

Type C (AR) is autonomous driving information superimposed on a real road scene, and virtual displays of lane lines and other traffic participants’ prompts.

### Scene complexity

**Fig 3:**
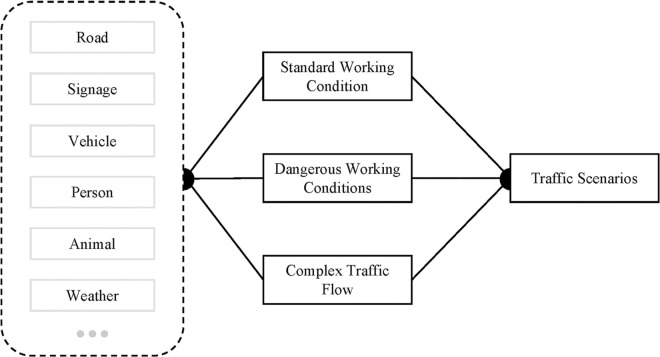
A path analysis model of the relationship between various scenario elements and traffic scenarios.

**Fig 4:**
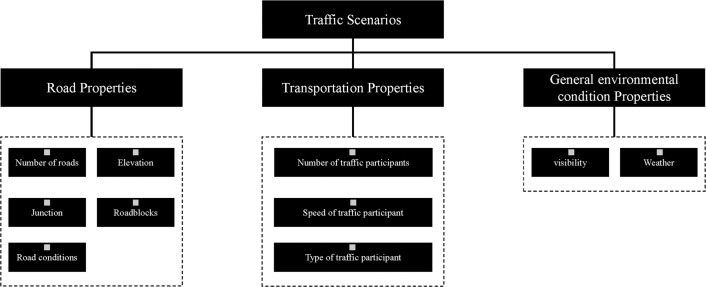
Conditional autonomous driving scenario complexity hierarchy.

The complexity of the scenario may have a bigger detrimental effect on driver safety, according to research, than the HUD’s actual design [26]. Three categories were created for the scene in this study using the scene system description model based on PEGASUS: road attributes, traffic attributes, and general environmental condition attributes, as shown in Fig 3. Also used are the publicly accessible datasets from the Wolfe (2020) study, which comprises 503 8-second movies shot from an automobile recorder’s point of view [27]. Considering the differences in road scenarios between China and the United States, the traffic signs, intersection designs, and driver interaction behaviors in the original scenarios have been adapted to local conditions to ensure the equivalence of the complexity assessment framework between the road environments of China and the United States. Based on the driving scenario images secondarily screened from the dataset, which include different weather conditions, seasons, and times on urban, rural, and highway sections, key elements are extracted to form scene factors, as shown in Fig 4, which complements and improves the framework of the complex element model of semi-autonomous driving scenarios.

When conditional autonomous driving scenarios are graded for complexity, factors such as the surrounding environment, road conditions, traffic flow, and driving conditions are combined to create a complicated driving environment. The Entropy Weight Method (EWM) is an objective weighting approach based on information entropy, which can integrate subjective with objective data. It determines weights by quantifying the information dispersion of indicators, providing an objective basis for decision-making analysis in multi-dimensional complex systems [28]. The principle is to utilize the amount of information provided by the indicator for weighting. For each given indicator, a higher entropy number indicates less discreteness, less information provided, less impact on the evaluation’s main goal, and a lower weight allocated to it. At present, scholars have applied EWM to the research of autonomous driving decision-making and driving scenarios. Pan (2022) employed the EWM to evaluate the operational characteristics of multi-lane turbo roundabouts and select optimal schemes [29]. Fu (2023) calculated a comprehensive weight coefficient for driving characteristic events through EWM, providing support for subsequent autonomous driving decisions [30]. To provide an effective basis for complexity analysis of conditional autonomous driving scenarios, this study adopted EWM to objectively determine the weight coefficients of various influencing factors in the driving scenario complexity evaluation system. Relevant experts were invited to score the importance of influencing factors, such as road conditions and traffic flow. The scoring values were then calculated to compute the scenario complexity level based on comprehensive indicators, resulting in conditional autonomous driving scenarios with different complexity levels.

A seven-point Likert scale was used to collect expert evaluations of the scenario elements. Ten experienced drivers were invited between May 20th, 2024 and May 22nd, 2024. This study strictly adhered to ethical guidelines, and the Ethics Committee of Nanjing Forestry University (Science and Technology Department of Nanjing Forestry University) has evaluated and authorized this protocol (Permit Number: 2024-05-16-11). All participants in this study were aware of its background, methodological procedures, results, and purpose, and had signed a written informed consent to participate in the study. In this study, drivers are classified as novices with less than 500 miles of driving experience and non-novices with more than 500 miles of driving experience [31]. The complexity judgment matrix is then built when the scene factor weights under the various elements are determined. After obtaining the expert rating data, it is necessary to construct a judgment matrix for the complexity weights of different factors, which is calculated as follows:

$$X^{*}=[\begin{array}{ccccc} X_{11}^{*} & \cdots & X_{1j}^{*} & \cdots & x_{1n}^{*}\\ X_{i1}^{*} & \cdots & X_{ij}^{*} & \cdots & x_{in}^{*}\\ X_{m1}^{*} & \cdots & X_{mj}^{*} & \cdots & x_{mn}^{*} \end{array}]$$ (1)

Where m is the number of evaluation objects, n is the number of evaluation factors and $X_{ij}^{*}$ is the value of the jth factor for the ith evaluation object. The judgment orthogonal matrix is also normalized as follows:

$$X^{i}=\frac{X_{ij}^{*}-\min X_{j}^{*}}{\max\{\begin{array}{c} X_{j}^{*} \end{array}\}-\min\{\begin{array}{c} X_{j}^{*} \end{array}\}}$$ (2)

Where ${\text{X}}^{\text{i}}$ is the normalized value in the matrix, i is the serial number of the evaluation object, and j is the serial number of the evaluation factor. The weight of the jth factor of the ith evaluation object is introduced and calculated as follows:

$$X_{ij}=\frac{X_{ij}^{*}}{\sum_{i=1}^{n}X_{ij}^{*}}$$ (3)

The information entropy value (*e*_*j*_), information entropy redundancy (*g*_*j*_), and factor weights (*w*_*j*_) of the jth factor are then calculated using Eqs (4)–(6) as follows:

$$e_{j}=K\sum_{i=1}^{n}p_{ij}\ln(\begin{array}{c} p_{ij} \end{array})$$ (4)

$$g_{j}=1-e_{j}$$ (5)

$$w_{j}=\frac{g_{j}}{\sum_{j=1}^{m}g_{j}}\;\;(\begin{array}{c} 1\leq j\leq m \end{array})$$ (6)

Where *k* > 0, $k=\frac{1}{\ln n}$, $e_{j}\geq 0$. The information entropy of each scene element component and the entropy weights of each design index are determined using Eqs (1)–(6). Table 1 displays the compiled results.

**Table 1:** Conditional autonomous driving scenario complexity factor perceptual weights.

Target layer	First level factor	Second level factor	Third level factor	Information Entropy(*H*_*j*_)	Weight(*W*_*j*_)
Scene complexity	Road Properties	Number of roads	Single Lane	0.927	1.955
			Multi-Lane	0.916	2.235
		Elevation	Gentle slope	0.884	3.112
			Steep slope	0.880	3.217
		Junction	Ingress and egress	0.917	2.216
			Intersection	0.828	4.600
		Roadblocks	Static roadblocks	0.880	3.217
			Dynamic roadblocks	0.803	5.252
		Road conditions	Flat	0.888	2.989
			Potholes	0.881	3.186
	Transportation Properties	Number of traffic participants	small number	0.929	1.906
			Numerous	0.861	3.708
		Speed of traffic participant	Low speed	0.918	2.203
			High speed	0.916	2.235
		Type of traffic participant	Pedestrian participants	0.927	1.955
			Small motor vehicle	0.759	6.434
			Large motor vehicles	0.673	8.734
			Non-motorized vehicles	0.907	2.492
	General environmental condition Properties	visibility	Low	0.579	11.259
			High	0.918	2.203
		Weather	Rain	0.673	8.734
			Snowy	0.759	6.436
			Haze	0.753	6.610
			Sunny	0.883	3.113

The detailed driving take-over scenarios are analyzed, and the complexity levels are determined using extensive metrics to produce conditional autonomous driving scenarios at various levels of complexity. We cluster three complexity scenario tiers: low, medium, and high. The low-level complexity scenarios are distinguished by safe driving conditions, minimal peri-vehicle components, and low traffic density. A greater number of peri-vehicle environmental elements, stationary impediments, and the risk of vehicle closeness due to obstacle avoidance are characteristics of scenarios with medium-level complexity. Enhanced traffic congestion, more environmental impediments, decreased environmental visibility, and dynamic, unpredictable obstacle features such as abrupt vehicle cut-ins and pedestrian intrusions are characteristics of high-level complexity situations.

### Participants

The sample size is important to the reliability and representativeness of the study findings. Therefore, the sample size needed to be estimated and determined before the experiment. Based on the effect size results from previous studies examining differences in SA test and eye movements, such as the stimulation of AR technology on subjects’ SA [32] and the stimulation of HUD colors on subjects’ eye movement data [33], and incorporating Cohen’s findings [34], sample size estimation was conducted using GPower software [35], specifically selecting the ANOVA: Repeated measures, within-between interaction type. The parameters included an alpha level of 0.05, a power level of 0.8, a medium effect size of 0.35, and a group count of 3. This setup yielded a total sample size requirement of 12 participants.

Eighteen young drivers were chosen to participate in this experiment (9 males: 5 novices, 4 non-novices; 9 females: 6 novices, 3 non-novices), with a male-to-female ratio of 1:1. They participated in the experiment from June 12th to June 18th, 2024. These subjects ranged in age from 24 to 29 years old on average (M = 23.85, SD = 3.91). This is because the younger generation constitutes the primary market for intelligent electric vehicles [36], and their driving behaviors and technological acceptance have direct reference significance for the design and R&D of autonomous driving in electric vehicles. A valid driver’s license and independent driving experience are essential requirements for all participants. When recruiting participants, the driving experiences of the participants were made equivalent to the extent possible. Every individual underwent experimental testing, was right-handed, and had normal or corrected vision. Participants in the study were free to discontinue participation at any time. In addition, this experiment strictly adhered to the ethical guidelines. Before participants engaged in the experiment, the procedures were thoroughly explained to them to ensure that the experiment would not pose any risks to the subjects, and written informed consent was obtained from all participants. The Ethics Committee of Nanjing Forestry University (Science and Technology Department of Nanjing Forestry University) has evaluated and authorized this experiment (Permit Number: 2024-05-16-10).

### Experimental design

To limit the impact of extraneous variables, including experimental order and grouping, on the experiment and to reduce statistical random errors, a 3×3 Latin-square design based on balanced experimental order was employed for experimental planning in this work. Two statistically non-interacting external variables were the type of autonomous driving information display and scene complexity. Each external variable was split into three equal levels for the Latin-square layout. Three distinct complexity levels (low-level, medium-level, and high-level) of the three displays (type A, type B, and type C) were investigated for their impact on drivers’ SA ability and take-over performance in Table 2. Furthermore, a variety of techniques (such as eye-movement data and SA questionnaires) were used to gather quantitative and qualitative information from each participant.

**Table 2:** 3x3 Latin square design.

Scene complexity	Experimental object		
	Group A	Group B	Group C
low-level	type A	type B	type C
medium-level	type B	type C	type A
high-level	type C	type A	type B

Since a 3rd-order Latin square design was adopted in this experiment, 18 subjects were divided into three groups, with 6 people in each group. They respectively carried out the simulation experiments of Group A, Group B, and Group C. In each group, the male-to-female ratio of the subjects was equal at 1:1, and the distribution of driving experience was basically similar.

#### Independent variables.

**Fig 5:**
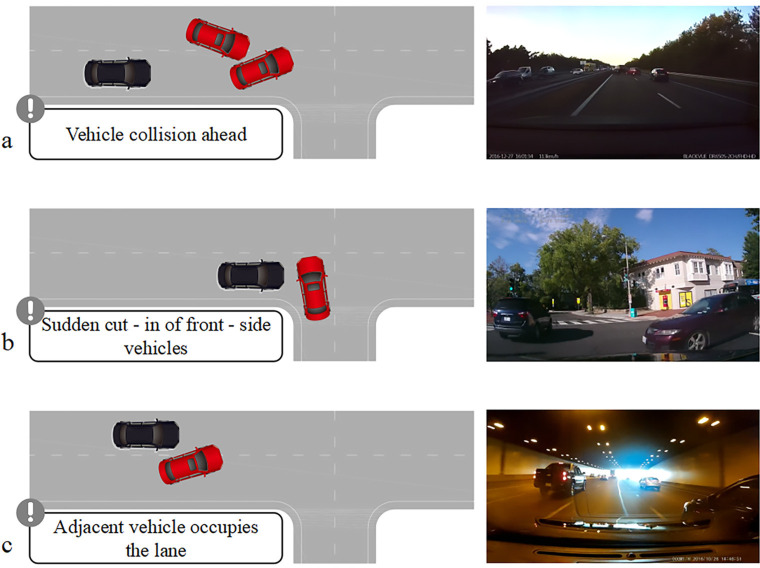
Three accident scenarios: a) forward vehicle collision; b) The sudden cut-in of lateral vehicles ahead; c) adjacent vehicle occupies the lane.

**Fig 6:**
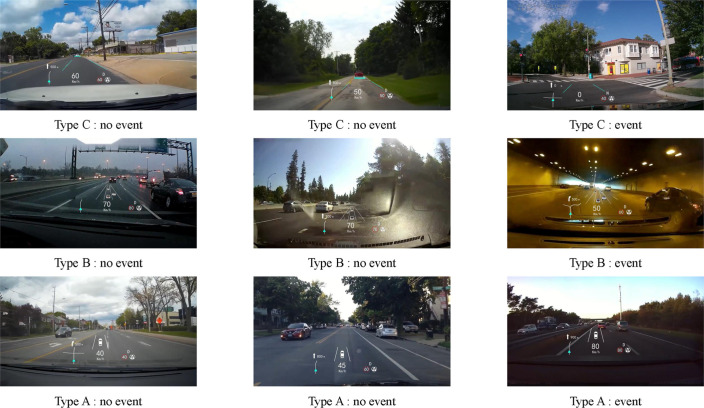
Example of Group C scene material.

In this investigation, two primary external independent factors were noted: the first was the kind of autonomous driving information display (type A, type B, and type C). The second was the driving scenario’s complexity (low-level, medium-level, high-level). Gender (male, female) was the between-subjects independent variable. From the driving scene video footage of Wolfe (2020), nine videos (including six no event videos and three event videos) were chosen based on the previously described forms of scene complexity [27]. In addition, the event video selects three of the most representative accident scenarios that require a driver to take over, including a forward vehicle collision, The sudden cut-in of lateral vehicles ahead, and an adjacent vehicle occupies the lane, as shown in Fig 5. They were divided into three separate scene complexity groups: Group A, Group B, and Group C. To avoid order effects, the material in each group’s various complexity scenarios was randomly arranged before the trial began. Fig 6 shows an example of Group C scene material. Adobe After Effects 2022 was used to attach three types of autonomous driving information, type A, type B, and type C, to the HUD display types for each group of nine video clips, while video watermarks were removed and driving audio was added to prevent interference with the subjects while providing them with an immersive autopilot experience.

#### Dependent variables.

The following sets of dependent variables were observed to provide answers to the two study hypotheses that were presented in the preceding part, eye-movement data and the SA questionnaire in the situational awareness portion, and take-over reaction time in the take-over performance section.

SA. For SA in non-take-over scenarios, the fixation duration of all gazes inside the Area of Interest (AOI) that corresponds to the location of the HUD element of each type of autonomous driving information display is the dependent variable [37]. The fixation duration refers to the time during which the foveal region of the visual field of the eye stays in the same place. During a longer fixation duration, there is a higher level of attention paid to that element. However, it may also indicate that this element imposes higher requirements on the cognitive abilities of the subjects [38,39]. The second dependent variable is the fixation counts within the AOI. The Situation Awareness Global Assessment Technique (SAGAT) score is the dependent variable for situational awareness in the take-over scenario [40]. Two questions were assigned to each of the three perceptual levels—sensing (SA-1), understanding (SA-2), and projecting (SA-3) that Endsley proposed. Example SA questions are provided in Table 3.

**Table 3:** SAGAT Assessment Sample Questions.

Perceptual Level	Question content
Perception (SA-1)	1. Which direction did you observr the traffic coming from? (Multiple choice)
	2. What is the speed limit you observed on this section of road? (Single choice)
Comprehension (SA-2)	3. Which direction did you judge to be dangerous, leading to your decision to take over the vehicle? (Single choice)
	4. Which direction do you need to drive at the next intersection? (Single choice)
Projection (SA-3)	5. Which other vehicle in which direction is closest to you when you decide to take over the vehicle? (Single choice)
	6. After you’ve gained control of the vehicle, what should you do next? (Single choice)

**Fig 7:**
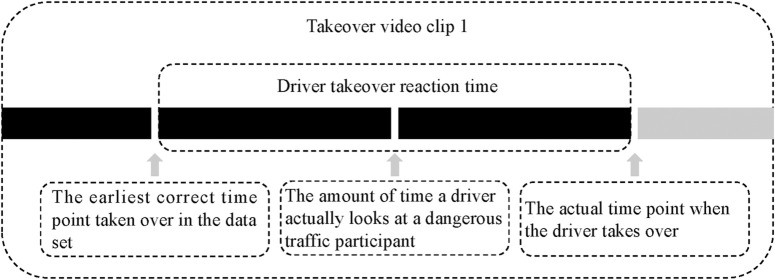
Schematic flow chart of driving take-over reaction experiment.

Take-over performance. The driving take-over reaction time, which was obtained from the eye movement data, served as the primary indicator of take-over performance in this investigation. The time difference between the take-over time point and the earliest proper time point of the take-over (the time point of the first noticeable departure from the cue) was primarily used to determine the driving take-over reaction time, as shown in Fig 7. In this experiment, clicking the mouse or pressing the space bar is recorded as a correct take-over (representing a hand movement).

### Experimental set-up

**Fig 8:**
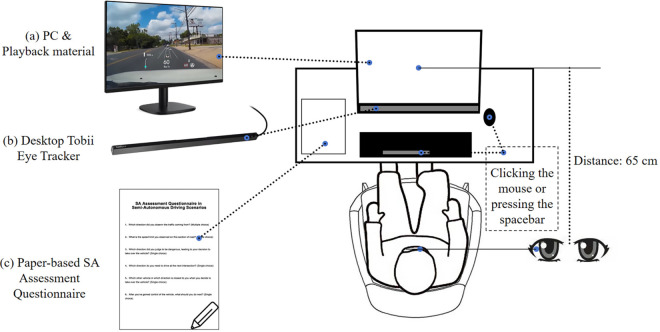
Experiment setting.

This experiment was carried out in a closed laboratory setting, free from outside distractions. The experimental setup for the study consisted of a set of desktop Tobii eye-trackers for human-computer interaction and psychology, manufactured by Tobii, Sweden, with a sampling frequency of 50 Hz and a gaze localization accuracy of 0.5. The mechanism was used to record the movements of the eye. To show the experimental simulations and collect experimental data, the Tobii Studio software was coupled and integrated with the ErGolab human-computer platform. The HP desktop PC running Windows 10 Professional and the HP 1680*1050 monitor were used to display the material stimuli. Both the basic interview and the SA questionnaire were completed on separate paper forms. During the experiment, participants sat approximately 65 centimeters away from the central screen and adjusted their viewing distance appropriately. Fig 8 shows a schematic diagram of the experimental setup.

### Procedure

**Fig 9:**
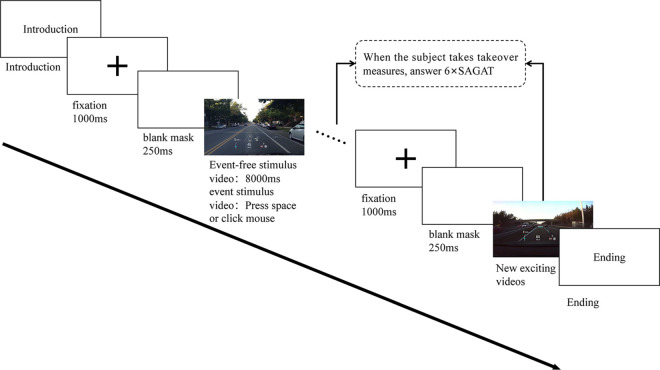
Experimental flow.

The experiment’s flow for the lone subject is depicted in Fig 9.

A questionnaire on their driving experience and fundamental data was sent out to those taking part. The goal of the experiment was fully explained to the subjects. Once they were fully aware of the procedure, participants were told to begin practicing. Each participant would practice before the official experiment until they felt at ease with the procedure. The exercises adhered to the same precise protocol as the formal experiment; the material that was delivered was the only variation.

Before beginning the experiment, the researcher checked to ensure each participant was in a suitable seated setting and understood the task. The next step consisted of a five-point binocular calibration. When the calibration was complete, it was indicated on a guide page. Once they understood what needed to be done for the task at hand, individuals were told to press the space button to continue. The stimulus material emerges after a 1000 ms fixed cross and a 250 ms blank mask. Participants were instructed to look at the center cross when the gaze cross appeared to prevent variations in eye gazing. Participants were permitted to see the content with several HUDs at the start of the stimulus. The experimenter paused the current experiment for a SA test of the frozen scene once the participant had gained control. Each stimulus was given for 8000 ms and could be discontinued at any point. The material for the next complexity scenario was obtained by the researcher with a mouse click once the participants had responded to the SA questions in a take-over scene. Every individual underwent the same lighting conditions and adhered to a standardized experimental protocol. The experiment took about twenty minutes to complete. After the experiment, the use of their experimental data and the protection measures will be explained to the participants.

## Results

All participants were sampled during eye movements in this experiment, and all experimental data from the final eighteen subjects were used for data analysis. To strictly protect the privacy of participants, all collected raw data were thoroughly anonymized before analysis. For details of the eye-tracking experiment data, please refer to the S1 File. The SPSS software version 25 was used for statistical analysis. Analysis of variance (ANOVA) was used to examine all the variables. Every multivariate test criterion in the results satisfied the same F-statistic. For all analyses, the effect sizes were measured using partial eta-squared ($\eta^{2}$), and the significance level was set at 0.05. Based on the Levene test, all data satisfied the chi-square and normal distribution assumptions. Using one or more of the reported variable dependents. The two study questions are the focus of this experimental investigation.

### Situational awareness

Because the participants’ take-over time points differed in the take-over scenario, potentially influencing the analysis’s findings, the eye movement data within the AOI was only used to examine driver situational awareness in the no-take-over scenario material. Under the take-over scenario material was analyzed only by studying the accuracy of the SAGAT questionnaire. Table 4 represents the mean (SDs) of fixation duration, fixation counts, and SAGAT test accuracy given by the independent variables for each scenario.

**Table 4:** Mean (SD) of fixation duration, fixation counts, and SAGAT accuracy according to scene complexity and autonomous driving information display type.

Display type	Scene complexity	No take-over				Take-over	
		Fixation Duration(s)		Fixation Counts		SAGAT test accuracy(%)	
		Male	Female	Male	Female	Male	Female
type A	low-level	2.58 (0.85)	2.71 (0.59)	8.00 (3.04)	5.67 (2.26)	0.72 (0.18)	0.61 (0.10)
	medium-level	0.75 (0.55)	0.69 (0.22)	2.83 (2.02)	3.33 (0.29)	0.75 (0.14)	0.67 (0.34)
	high-level	3.77 (0.24)	1.37 (0.38)	6.33 (0.29)	4.17 (0.28)	0.64 (0.27)	0.64 (0.05)
type B	low-level	4.39 (1.38)	2.86 (1.86)	5.17 (1.76)	6.83 (2.02)	0.70 (0.05)	0.44 (0.20)
	medium-level	2.04 (1.21)	2.31 (0.94)	9.33 (5.01)	6.67 (1.04)	0.61 (0.17)	0.56 (0.10)
	high-level	1.87 (1.08)	1.35 (0.48)	4.50 (1.80)	5.83 (2.02)	0.28 (0.05)	0.19 (0.17)
type C	low-level	3.10 (1.83)	2.40 (1.00)	5.50 (0.00)	6.83 (1.26)	0.92 (0.14)	0.94 (0.10)
	medium-level	1.96 (1.81)	0.73 (0.64)	2.67 (1.53)	3.33 (3.01)	0.80 (0.05)	0.80 (0.05)
	high-level	0.73 (0.39)	1.26 (0.69)	2.33 (1.26)	3.00 (1.32)	0.92 (0.14)	0.75 (0.25)

#### Fixation duration.

**Fig 10:**
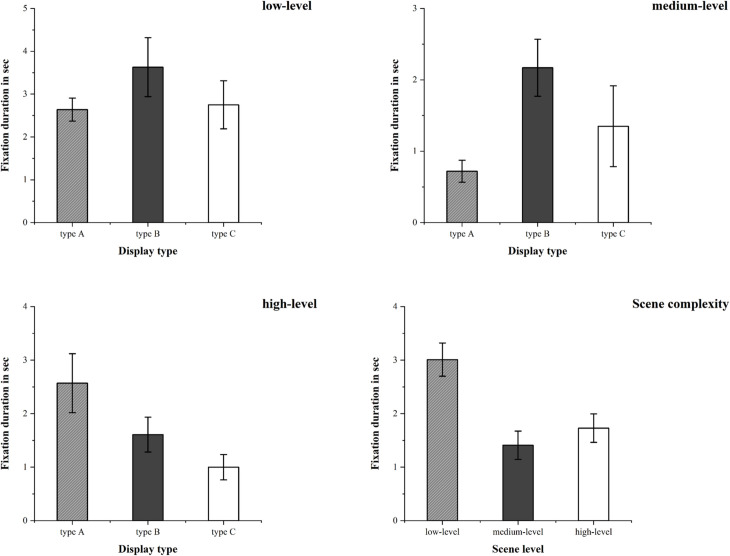
The mean and standard error of fixation duration for type A, type B, and type C autonomous driving information display types under various scenario complexities.

When comparing the fixation duration of participants on different types of autonomous driving information displays, as shown in Fig 10, the effects of gender [F(1,36) = 4.74, p = 0.036, $\eta^{2}$ = 0.116] and scenario complexity [F(2,36) = 11.94, p = 0.000, $\eta^{2}$ = 0.399] were found to be the most significant. The data showed that the fixation duration of males was significantly longer than that of females. Compared with medium and high-complexity scenarios, participants spent significantly more fixation duration on autonomous driving information in low-complexity scenarios.

Additionally, the interaction effect between information display type and scenario complexity was also significant [F(4,36) = 2.77, p = 0.042, $\eta^{2}$ = 0.236]. When fixing the scenario complexity, in medium-complexity scenarios, the fixation duration of type A was significantly shorter than that of type B. In high-complexity scenarios, the fixation duration of type C was significantly shorter than that of type A.

When fixing the display type, for type A, the fixation duration in medium-complexity scenarios was significantly lower than that in low and high-complexity scenarios. For type B, the fixation duration in high-complexity scenarios was significantly higher than that in low and medium-complexity scenarios. For type C, the fixation duration in low-complexity scenarios was significantly higher than that in medium and high-complexity scenarios.

#### Fixation counts.

**Fig 11:**
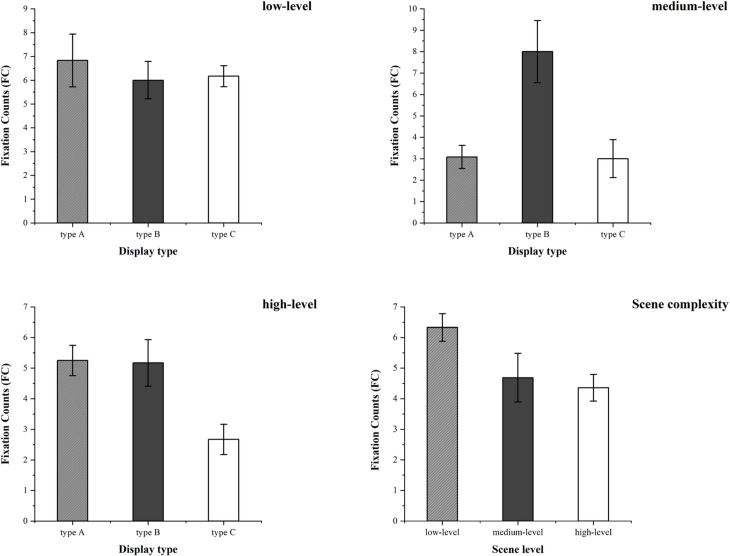
The mean and standard error of fixation counts for type A, type B, and type C autonomous driving information display types under various scenario complexities.

When comparing the fixation counts of participants on different types of autonomous driving information displays, as shown in Fig 11, the effects of scenario complexity [F(2,36) = 4.77, p = 0.015, $\eta^{2}$ = 0.21] and display type [F(2,36) = 6.41, p = 0.004, $\eta^{2}$ = 0.263] were observed to be the most significant. The data showed that low-complexity scenarios attracted more fixation counts than medium and high-complexity scenarios.

Additionally, the interaction effect between information display type and scenario complexity was also significant [F(4,36) = 4.32, p = 0.006, $\eta^{2}$ = 0.324]. When fixing the scenario complexity, in medium-complexity scenarios, type B attracted more fixation counts than type C and type A. In high-complexity scenarios, type C attracted fewer fixation counts than type A and type B.

When fixing the display type, for type A, the fixation counts in medium-complexity scenarios was significantly fewer than that in low-complexity scenarios. For type B, the fixation counts in medium-complexity scenarios was significantly more than that in high-complexity scenarios. For type C, the fixation counts in low-complexity scenarios was significantly more than those in medium and high-complexity scenarios.

#### SAGAT accuracy.

**Fig 12:**
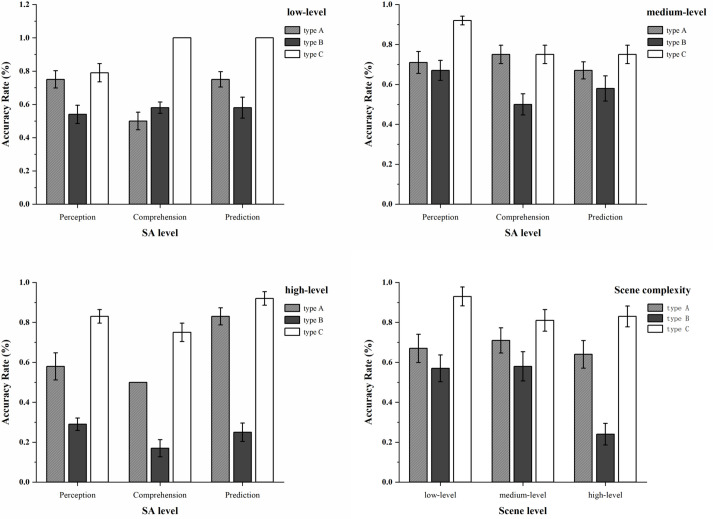
The mean and standard error of SAGAT accuracy for type A, type B, and type C autonomous driving information display types under various scenario complexities.

When comparing the SAGAT level questionnaire measurement results of participants on different types of autonomous driving information displays, as shown in Fig 12, the results showed that scenario complexity [F(2,36) = 6.07, p = 0.005, $\eta^{2}$ = 0.252], display type [F(2,36) = 34.08, p = 0.001, $\eta^{2}$ = 0.654], and gender [F(1,36) = 4.21, p = 0.048, $\eta^{2}$ = 0.105] had the most significant effects on SA accuracy. The data showed that participants’ SA accuracy in low-complexity scenarios was significantly higher than that in medium and high-complexity scenarios. Additionally, their SA accuracy for type C was significantly higher than that for type A and type B. Furthermore, males had significantly higher SA accuracy than females.

Additionally, the interaction effect between information display type and scenario complexity was also significant [F(4,36) = 3.57, p = 0.015, $\eta^{2}$ = 0.284]. When fixing the scenario complexity, in low-complexity scenarios, type C had higher SA accuracy than type A and type B in terms of comprehension and prediction. In medium-complexity scenarios, there was no significant difference among the three display types. In high-complexity scenarios, both type A and type C were significantly better than type B in the three aspects of perception, comprehension, and prediction.

### Take-over performance: take-over reaction time

The data labeled in the public database by Wolfe (2020) was used as a reference to determine the earliest correct time point of take-over for the take-over material at varied scene complexities (Table 5) [27]. Table 6 displays the mean (SDs) of the computed take-over reaction times for the six participants for each group of experiments after the take-over reaction times for each group in the experimental data have been arranged.

**Table 5:** Point in time of the first visible deviation from the trail in each scenario.

Scene complexity	First Visible Cue of Deviation (s)	Description of hazard
low-level	5.33	The vehicle runs a red light
medium-level	5.5	The leading vehicle collides with the vehicle ahead
high-level	3.5	A vehicle in the right lane drifts into the lane of travel

**Table 6:** Mean (SDs) of take-over reaction times according to scene complexity and type of autonomous driving information display.

Display type	Scene complexity	take-over reaction time(s)	
		Male	Female
type A	low-level	0.80 (0.25)	0.54 (0.23)
	medium-level	1.20 (0.54)	0.74 (0.25)
	high-level	0.48 (0.03)	1.07 (0.12)
type B	low-level	0.93 (0.41)	1.79 (1.00)
	medium-level	1.39 (0.57)	2.07 (0.82)
	high-level	0.69 (0.20)	0.79 (0.71)
type C	low-level	0.54 (0.37)	1.25 (0.98)
	medium-level	0.66 (0.31)	0.88 (0.40)
	high-level	0.44 (0.26)	0.75 (0.22)

**Fig 13:**
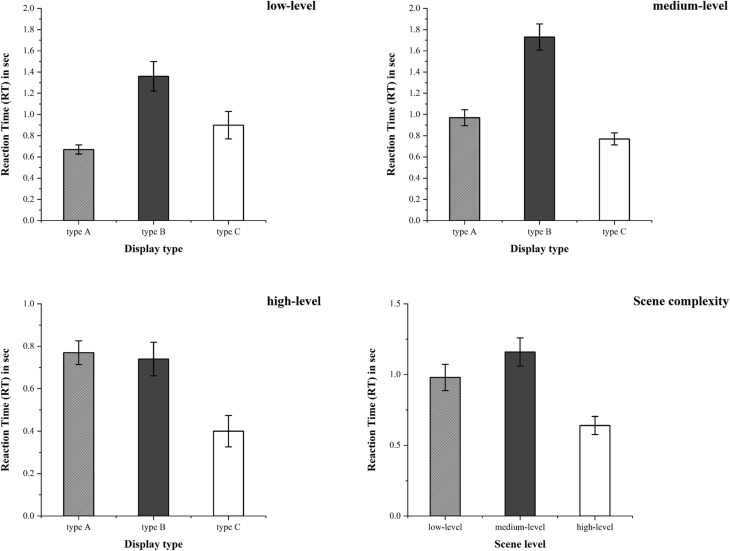
The mean and standard error of take-over reaction time for type A, type B, and type C autonomous driving information display types under various scenario complexities.

When comparing the take-over reaction time results of participants for different types of autonomous driving information displays in each take-over scenario, as shown in Fig 13, it was observed that gender [F(1,36) = 5.24, p = 0.028, $\eta^{2}$ = 0.127], display type [F(2,36) = 6.941, p = 0.003, $\eta^{2}$ = 0.278], and scenario complexity [F(2,36) = 5.827, p = 0.006, $\eta^{2}$ = 0.245] had the most significant effects on take-over reaction time. Additionally, no significant interaction effect was found among participants’ gender, display type, and scenario complexity.

The data showed that in terms of scenario complexity, participants’ take-over reaction time in high-complexity scenarios was significantly shorter than that in low and medium-complexity scenarios. In terms of display type, there was a significant difference in take-over reaction time between type B and type C in medium-complexity scenarios. In other scenarios, there was no significant difference among type A, type B, and type C. In terms of gender, for type B and type C, male participants had significantly faster take-over reaction time than females.

## Discussion

This work aims to determine the optimal display types of autonomous driving information on the HUD suitable for young drivers in different driving environments, and provide insights for the design of autonomous driving information on the HUD. When considering the two research questions in the introduction, the experiment yielded some interesting findings.

The eye tracking method revealed that under normal driving conditions, drivers paid less visual attention to the AR presentation of information in situations with different levels of complexity. Moreover, the take-over scenario demonstrated that in all three complexity settings, the accuracy of AR-type’s SA reporting was generally higher. The majority of the study’s conclusions are in line with earlier investigations. Researchers in a variety of industries have demonstrated how AR display-style interfaces can enhance users’ SA. For instance, Rowen (2019) discovered that AR devices can considerably enhance users’ situational awareness abilities in dynamic contexts, including SA-1 and SA-2 [41]. The current study’s findings partly agree with those of earlier research conducted in various complexity scenarios. A variety of intricate scenarios could be the cause of this. The results of a study showed that the level of SA during night driving was significantly improved compared with that during daytime driving, while the improvement of the SA level during daytime driving was not significant. This demonstrates the different effects that changes in environmental complexity can have on drivers’ SA levels [42]. Therefore, we found that the lower the complexity of the scene was, the smaller the difference in the impact on drivers between AR display and simple P3D display would be, conversely, a better performance of AR display is achieved. Meanwhile, the SR display effect is the worst of the three display kinds. A study found that when objects approach at different distances from the observer but with the same speed and trajectories, it can be challenging for the observer to determine the relative speeds of the two objects, among other things [43]. This explains why drivers must exert greater mental effort to appraise other traffic participants in SR displays when they are shown in front of them at the same speed and trajectory as the SR automobile model on the HUD.

It was discovered that AR displays outperformed P3D and SR displays in terms of driver take-over performance across all complexity levels, particularly in high-complexity scenarios. This result is in line with the findings of You (2024) who showed that drivers’ reaction times in both human-driven and autopilot modes can be greatly shortened by AR display information and that this can increase human drivers’ confidence in the autopilot system [44]. Furthermore, the current study discovered that the driver’s take-over performance rose with scenario complexity, irrespective of the type of autonomous driving information display that the driver encountered. This performance is consistent with earlier research on driving, such as Zhang (2013) who observed that drivers’ safety margins and speeds were more conservative as road complexity rose, indicating that they were also paying more attention [45]. It can therefore be inferred that the AR display is the best option for the current type of autonomous driving information display in various complexity scenarios. However, further research is needed to investigate the more specific expressions of autonomous driving information in the AR interface.

Meanwhile, in addition to the findings regarding the study’s two research questions, this experiment has also yielded some interesting additional discoveries concerning the gender factor. Previous research has shown that the visual colors of HUD interfaces produce differences in gender perception [33]. Since all HUD types display the same visual of autonomous driving information to the driver in this experiment, potential differences in perception of different display types by different genders in scenarios of varying complexity could be observed. Eye-tracking techniques have revealed that males always have higher visual attention than females for autonomous driving information in HUD. Furthermore, males are always faster than females in take-over. This may share some similarities with the findings of Loeb (2019) [46]. In their study, when an autonomous vehicle unexpectedly drove toward a closed highway exit, the crash rate among males (38%) was lower than that among females (43%). This indicates that in emergency take-over scenarios, male drivers exhibit higher take-over efficiency than female drivers. This may be related to the significant differences they exhibited at the SA-2 and SA-3 levels. Simultaneously, SR displays have the greatest visual attraction for both males and females, showing that both males and females are not suitable for using SR displays. These findings show that gender may be an important factor affecting autonomous driving information processing and response, which is worthy of further exploration. However, due to the limitations and specificity of the sample size in this study, more experiments are needed to obtain gender-based perceptual differences in the display types of autonomous driving information on HUD.

To sum up, our findings indicate that the most effective ergonomic option for enhancing the young driver’s take-over performance and SA skills is AR-displayed autonomous driving information, particularly in cases with a high degree of complexity. However, not every car has AR-HUD as standard equipment (e.g., AR-HUD is a premium option on the Volkswagen ID4 models). As a result, according to the results of this study, on the W-HUD, it is recommended to use a P3D type to display autonomous driving information.

There are some limitations to this study. First, one limitation of this study is that the experiment was conducted in a laboratory environment. Although the experimental setup was designed based on the results of previous studies [47,48], and to enhance the immersion and realism for participants during the experiment, this study used real-world recorded driving videos that met the requirements (the video materials were designed to cover diverse road features and climatic conditions as much as possible), rather than simulated scenarios. But the limitations of the laboratory and equipment make it impossible to capture all the characteristics of the natural driving environment, nor can they fully replicate the unpredictable risks and drivers’ psychological pressures in real driving. This limits the realization of a more realistic driving simulation experience and also makes the conclusions of this study mainly applicable to controlled dynamic scenarios similar to the experimental conditions. For the application of the conclusions in real environments, future studies should explore the effects of different autonomous driving information displays on the HUDs in actual driving environments to collect more precise and objective data (including verification across a broader range of regions and climates). Furthermore, misjudging the driver’s position of other traffic participants can result from the FOV of the HUD’s optical imaging deviating from the driver’s perceived position [49], a factor that was overlooked in this investigation.

The other main limitation of this study is that although we demonstrated the three main types of autonomous driving information display on the HUDs available in the market in the experiment and their performances in scenarios with different levels of complexity, we did not discuss in detail the specific HUD information design schemes, nor did we propose entirely new display principles or interaction models. Future work can build on the results of this study to explore new interaction mechanisms. Meanwhile, the impact of merging various autonomous driving information display formats on car HUD (e.g., the AITO M9 model’s combination of AR and SR displays) might also be studied further.

Finally, the participants in this study were concentrated in the young group aged 24 to 29. Although this group represents the main consumer segment of the current electric vehicle market in most regions, limitations in their driving experience, risk perception, and other characteristics may fail to reflect the performance of drivers of other age groups. This limitation may restrict the generalizability of the conclusions to electric vehicle drivers across all age groups. Future studies can include samples from a broader range of age groups to enhance the generalizability of the findings. Meanwhile, comparing the differences in how drivers of different age groups process autonomous driving information on the HUD is another research direction worthy of further exploration through corresponding experimental designs.

## Conclusion

The purpose of this study was to examine the effects of different autonomous driving information display types on HUD that are currently on the Chinese market on participant take-over efficiency and SA under scenarios with different levels of complexity. Based on the existing solutions in the market, three representative display types of autonomous driving information on the HUD were extracted and designed. The EWM was used to classify the semi-autonomous driving scenarios’ complexity into low, medium, and high levels. An eye-movement-based experiment was carried out to assess the effects of three different types of display schemes and three different complexity scenarios on the driver. The AR-HUD has the best potential to increase driver safety awareness when compared to the P3D and SR display formats on the W-HUD, and this advantage will be strengthened as the complexity of the scene increases. Furthermore, in high-complexity scenarios, the driver take-over is more efficient than in low-complexity scenarios, and males are always faster than females in taking over. For autonomous driving information in the HUD, males always pay more visual attention than females, but both males and females are more suitable for using AR display and P3D display. Before applying the ideal autonomous driving information display scheme to car HUD design, designers must take into account the driving environment of the vehicle, the type of HUD, and the gender of the drivers.

The study’s findings can be used to improve the decision-making on the display types of autonomous driving information on HUD based on different levels of scenario complexity similar to those in this experiment. This may enable different autonomous driving information display types to be displayed more effectively in relation to various scenario elements, lessening user cognitive load and saving the company money on development. The aforementioned results have implications for enhancing the autonomous driving information’s SA in HUDs. In the future, concerning the general laws and complexity issues of appropriate autonomous driving information display types in controlled scenarios with different complexities summarized from the experiments, the experimental procedures can be further enhanced and varied in more diverse scenario experiments to expand the applicable scope of the research.

## Supporting information

S1 File Supplementary raw data file of eye-tracking experiment for subjects. (XLSX)
